# *p16^INK4a^* Suppression by Glucose Restriction Contributes to Human Cellular Lifespan Extension through SIRT1-Mediated Epigenetic and Genetic Mechanisms

**DOI:** 10.1371/journal.pone.0017421

**Published:** 2011-02-24

**Authors:** Yuanyuan Li, Trygve O. Tollefsbol

**Affiliations:** 1 Department of Biology, University of Alabama at Birmingham, Birmingham, Alabama, United States of America; 2 Center for Aging, University of Alabama at Birmingham, Birmingham, Alabama, United States of America; 3 Comprehensive Cancer Center, University of Alabama at Birmingham, Birmingham, Alabama, United States of America; 4 Nutrition Obesity Research Center, University of Alabama at Birmingham, Birmingham, Alabama, United States of America; 5 Comprehensive Diabetes Center, University of Alabama at Birmingham, Birmingham, Alabama, United States of America; National Institute on Aging (NIA), National Institutes of Health (NIH), United States of America

## Abstract

Although caloric restriction (CR) has been shown to increase lifespan in various animal models, the mechanisms underlying this phenomenon have not yet been revealed. We developed an *in vitro* system to mimic CR by reducing glucose concentration in cell growth medium which excludes metabolic factors and allows assessment of the effects of CR at the cellular and molecular level. We monitored cellular proliferation of normal WI-38, IMR-90 and MRC-5 human lung fibroblasts and found that glucose restriction (GR) can inhibit cellular senescence and significantly extend cellular lifespan compared with cells receiving normal glucose (NG) in the culture medium. Moreover, GR decreased expression of *p16^INK4a^* (*p16*), a well-known senescence-related gene, in all of the tested cell lines. Over-expressed *p16* resulted in early replicative senescence in glucose-restricted cells suggesting a crucial role of *p16* regulation in GR-induced cellular lifespan extension. The decreased expression of *p16* was partly due to GR-induced chromatin remodeling through effects on histone acetylation and methylation of the *p16* promoter. GR resulted in an increased expression of SIRT1, a NAD-dependent histone deacetylase, which has positive correlation with CR-induced longevity. The elevated SIRT1 was accompanied by enhanced activation of the Akt/p70S6K1 signaling pathway in response to GR. Furthermore, knockdown of SIRT1 abolished GR-induced *p16* repression as well as Akt/p70S6K1 activation implying that SIRT1 may affect *p16* repression through direct deacetylation effects and indirect regulation of Akt/p70S6K1 signaling. Collectively, these results provide new insights into interactions between epigenetic and genetic mechanisms on CR-induced longevity that may contribute to anti-aging approaches and also provide a general molecular model for studying CR *in vitro* in mammalian systems.

## Introduction

A growing body of evidence indicates that restricted caloric intake is associated with lifespan extension and longevity in various organisms including yeast, worms, flies and even mammals [1]–[5]. At the physiological and pathological levels, caloric restriction (CR) can prevent the onset of many age-related degenerative diseases such as cancer, cardiovascular disease and diabetes in experimental animal models and human populations [6]. Hence, CR is by far the most effective environmental manipulation to extend maximum lifespan. Potential molecular mechanisms by which CR induce longevity may involve oxidative or metabolic pathways and more likely regulation changes of various age-related genes [7]–[9]. However, the precise mechanisms of CR-induced lifespan extension and longevity are not very well understood. Therefore, finding the molecular mechanisms whereby CR regulates lifespan has attractive potential in aging studies.

Epigenetic events are among the most striking mechanisms responsible for nutrition-related longevity, which is believed to dynamically regulate gene expression by primarily impacting two epigenetic codes, DNA methylation and histone modification [10]–[12]. As evidence of this, the yeast protein silent information regulator 2 (Sir2), a nicotinamide adenine dinucleotide (NAD^+^)-dependent histone deacetylase (HDAC), is a key determinant in CR-induced lifespan prolongation in yeast [5], [13]. In mammalians, SIRT1 is one of seven mammalian orthologs of Sir2, which has been extensively studied for its roles in chromatin remodeling and lifespan elongation. SIRT1 acts as a nutrient sensor involved in the regulation of various gene expressions as well as modulation of important signal transductions either directly or indirectly through its unique epigenetic effects, which ultimately influence the regulation of longevity [14]. Our previous studies indicated that glucose restriction-induced DNA methylation alteration in the *p16* promoter contributes to cellular lifespan extension [15]. In this regard, epigenetic mechanisms are major molecular events which play a crucial role in CR-induced longevity. Therefore, we speculated that an aging-associated gene such as *p16* may have a central position in epigenetic control of inhibition of cellular senescence and lifespan elongation in response to CR.

The *p16^INK4a^* gene, a cyclin-dependent kinase inhibitor, is believed to play an important role in tumor growth suppression and cell senescence [16], [17]. The accumulation of *p16* contributes to senescence by negatively regulating the cell cycle *in vitro* and *in vivo* [18], [19]. In addition, *p16* is also an epigenetic-regulated gene, since its expression is frequently modulated by epigenetic processes [20], [21]. Further, our previous studies suggested that the accumulation of *p16^INK4a^* was attenuated by glucose restriction in normal human lung fibroblasts in part by epigenetic control but not repressed in precancerous fibroblasts of the same origin [15]. We therefore sought to investigate the molecular mechanisms of epigenetic modulation of *p16* expression, which will facilitate approaches to anti-aging and anti-carcinogenesis studies.

Although the effect of CR in animal models is obvious, some studies have revealed that the effects of CR-inducing longevity in experimental animal models have varied, which may be due to genetic variation between different species as well as different living conditions for experimental animals [22], [23]. Therefore, these uncontrolled factors in CR animal models may reduce its utility in mechanisms studies. However, a novel *in vitro* cellular system for CR has more advantages such as flexible-control, accuracy and identical genomic background as compared to the *in vivo* systems. This system allows more precise analysis of molecular mechanisms of CR specifically at the cellular level in addition to the effects of CR on cellular lifespan. Previously we established an *in vitro* system to mimic CR-controlled longevity by reduction of glucose, the main caloric resource, in cell culture medium [15]. Hence we have extended our studies to elucidate fundamental epigenetic mechanisms in regulating cellular lifespan in three human normal fibroblasts in response to glucose restriction (GR). We found that GR can extend cellular lifespan through inhibition of the accumulation of *p16*. Perhaps most importantly, this is due at least in part to epigenetic modulation of *p16* expression through SIRT1-dependent and -independent histone remodeling. Our results also showed that SIRT1 can regulate Akt/p70S6K1 signaling that contributes to *p16* suppression leading to longevity in GR. This implies a novel crosstalking mechanism involving epigenetic and genetic control of *p16* regulation and also suggests that SIRT1 has a key position in connecting of these two mechanisms. Our findings not only reveal CR-involved epigenetic mechanisms in cellular lifespan control, but also provide new insights into nutrition-related anti-aging and anti-cancer approaches.

## Results

### GR delayed cellular senescence processes leading to cellular lifespan extension in normal human fibroblasts

**Figure 1:**
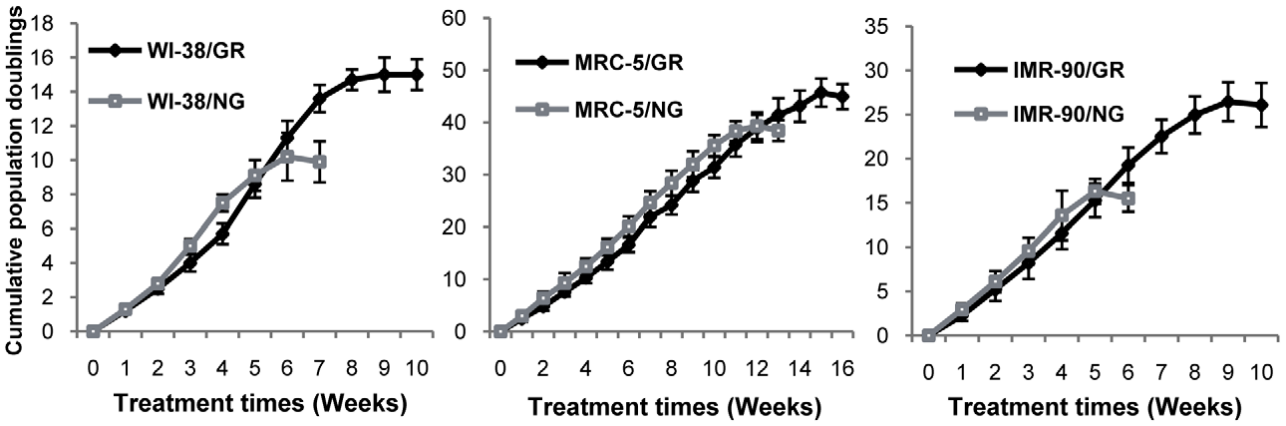
Glucose restriction extends lifespan of human fibroblasts. Cumulative population doubling curves of WI-38 (left), MRC-5 (middle) and IMR-90 (right) in either normal glucose (NG) level or glucose restriction (GR) growth medium. Viable cells were counted weekly by trypan blue staining using a hemacytometer. Population doublings were calculated by the formula log [(number of cells harvested)/(number of cells seeded)]/log2. Each graph depicts the averaged results from three longevity assessments.

Our previous studies indicated that GR can increase the lifespan of normal WI-38 fibroblasts. It is very important to verify that this phenomenon occurs not only in a single cell type, but also in multiple cell models. We therefore extended this study by including two fetal lung fibroblasts such as MRC-5 and IMR-90 as well as WI-38 that we employed in our previous studies [15] to allow multiple cell comparisons and further mechanistic analysis. To restrain energy intake, we treated the fibroblasts with glucose restriction (GR) medium, whereas the same batches of fibroblasts treated with normal glucose (NG) level of medium served as control. We monitored the cellular proliferation and population doublings (PDs) during the entire cellular lifespan in three fibroblast cell lines receiving either NG or GR medium. Experiments were terminated when cells stopped proliferating and underwent replicative senescence. As indicated in Fig. 1, the lifespan of all three human fibroblasts cell lines grown in GR medium was extended by an additional 2–4 weeks accompanied by an additional 5–10 PDs, which constituted a 23–67% prolongation of lifespan depending on different types of cells in GR medium (Table 1). Notably, this extension did not proceed prominently at the beginning of GR treatment, but rather, was more noticeable during the late treatment of GR. For instance, in WI-38 cells, a slightly reduced population doubling was observed at the beginning of four week-treatment of GR. After that, glucose-restricted WI-38 cells surpassed the maximal lifespan and underwent a total expansion of lifespan by more than four weeks with an additional 5.2±0.3 PDs (Fig. 1, left panel and Table 1) compared with WI-38 cells in NG medium. These results indicate that cells may need an adaptive process to undergo longevity under the situations of GR or caloric restriction (CR) [6], [7]. Consistent with our previous studies, this study further established that CR can induce longevity not only in experimental animal models, but more importantly, in a culture cellular system, which may provide an excellent *in vitro* model for CR studies. It is also notable that although GR extended the lifespan of all of the cell lines we examined, this varies somewhat for different cells.

**Table 1:** Extension of population doublings and cellular lifespan in response to glucose restriction.

	Cumulative population doublings#			Lifespan
Cell lines	Glucose	Glucose restriction	Extension	Percentage of extension (%)
**WI-38**	9.9±1.2	15.1±0.9	5.2±0.3	42.8
**MRC-5**	38.4±2.0	44.9±2.4	6.5±0.4	23.1
**IMR-90**	15.5±1.5	26.1±2.5	10.6±1.0	66.7

# The cumulative population doublings were calculated after the first confluent cells designed as PD 0.

**Figure 2:**
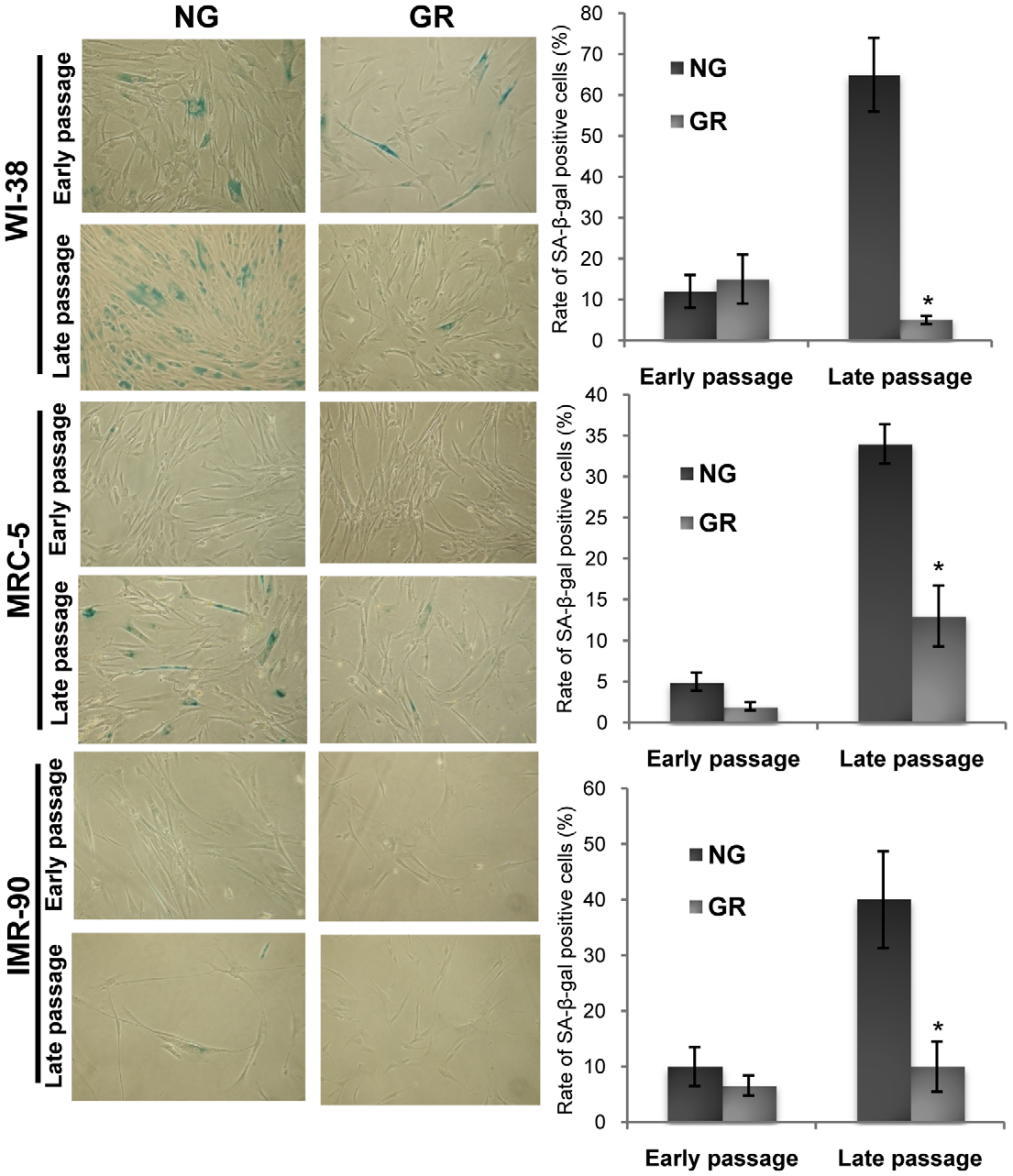
GR delayed cellular senescent rate. Cellular senescence was determined by Senescence β-Galactosidase (SA-β-Gal) assay. Human fibroblasts, WI-38 (upper), MRC-5 (middle) and IMR-90 (lower) treated with NG and GR growth medium were subjected to SA-β-Gal staining and photographed in a proliferating state (early passage) and senescence (late passage). The definitions of early and late passages were mentioned in the section of Experimental procedures. Magnification, ×100. The rate of SA-β-Gal-positive cells (blue staining) was counted by the number of SA-β-Gal-positive cells divided by the total number of cells in 5 randomly chosen fields. The graphs (right) shown are representative of the results obtained from three independent experiments. Bars, SD; *, *P*<0.05, significantly different from NG control.

To determine the effect of GR on cellular senescence, we quantified the proportion of senescent cells at their early and late passages under the condition of either NG or GR medium by using the senescence-associated β-galactosidase (SA-β-gal) activity assay. We found a low proportion of senescent cells in all three types of fibroblasts at the early stage of cell proliferation both in NG and GR medium-treated cells (Fig. 2). Further, no prominent differences in SA-β-gal activity were observed between fibroblast cells with the treatment of NG and GR medium at early passage. However, successive subculture resulted in significantly increased senescent cells accumulation in NG medium *versus* GR medium at late passage. This replicative senescence delay induced by GR occurred at late passage rather than at early passage further indicating a potential cellular metabolic adaptive process as discussed above. Taken together, our results suggest that GR significantly extends the lifespan of multiple cell lines through suppressing cellular aging processes leading to a prolongation of lifespan *in vitro*.

### GR influenced cellular senescence through inhibition of *p16* expression

*p16^INK4a^* (*p16*) is well known as a negative regulator of the cellular cycle through inhibiting activation of cyclin D/CDK4/CDK6 and subsequently releasing the inhibitory retinoblastoma gene (Rb) [24]. More importantly, significant accumulation of *p16* expression has been widely observed in a variety of aging tissues both in animal models and humans indicating *p16* plays an important role in aging and can also serve as a potent aging biomarker. Considering the important roles of *p16* in aging, we sought to define the patterns of *p16* expression in normal human cells during GR treatment.

**Figure 3:**
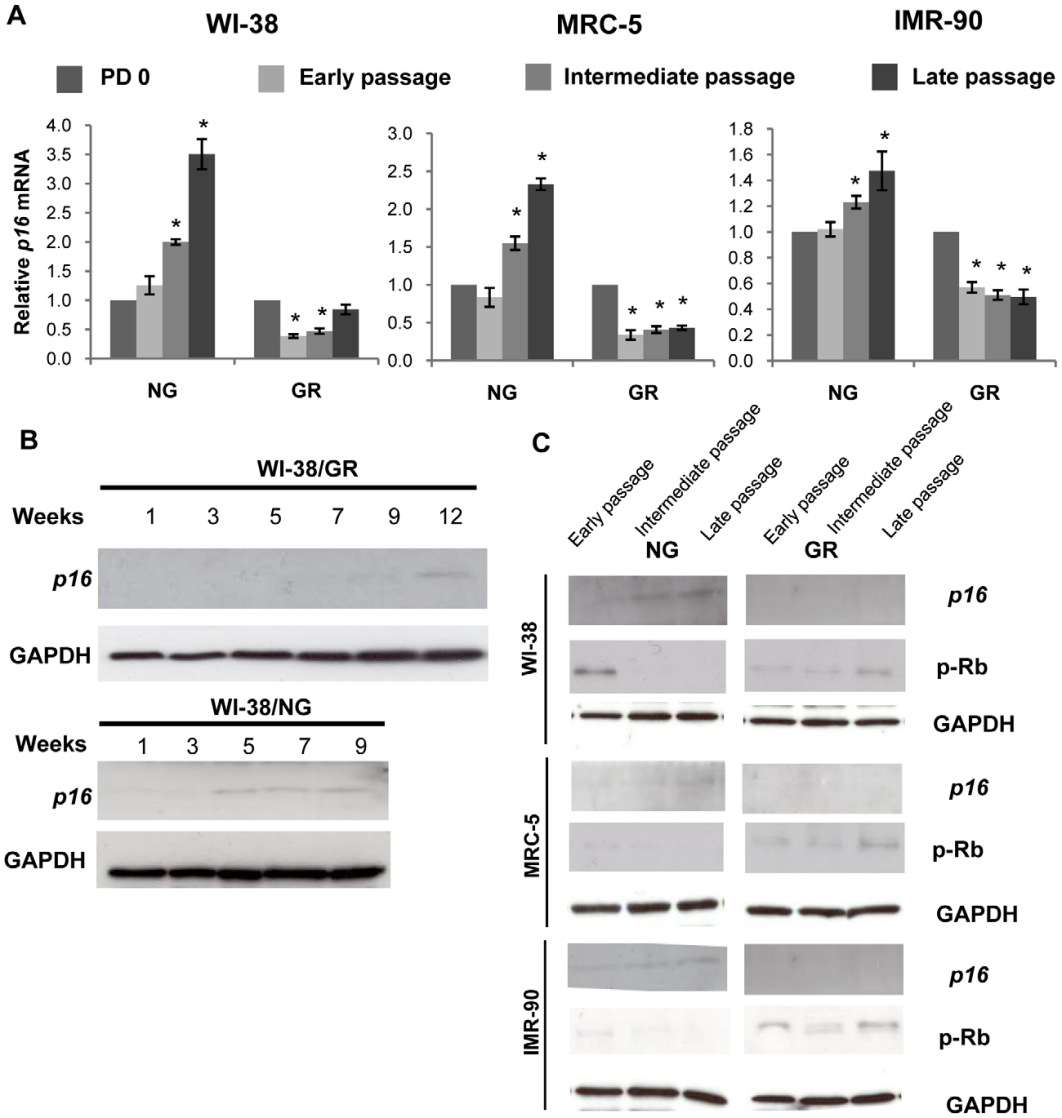
GR resulted in *p16* repression. A. Graphic presentation of relative mRNA levels of *p16* in WI-38 (left), MRC-5 (middle) and IMR-90 (right) cells during early, intermediate and late proliferation of cell growth. Human fibroblasts were cultured in NG or GR medium as indicated previously. Data are in triplicate from three independent experiments and were normalized to GAPDH. *p16* mRNA level at PD 0 was calibrated to 1. Columns, mean; Bars, SD; *, *P*<0.05, significantly different from *p16* mRNA level at PD 0. B. The protein levels of *p16* were determined by western-blot analysis. WI-38 cell proteins were extracted once every two weeks until cells ceased growth and underwent replicative senescence. C. The protein expression of *p16* and phosphorylated Rb (p-Rb) were detected in human fibroblasts, WI-38 (upper), MRC-5 (middle) and IMR-90 (lower), during early, intermediate and late proliferation of cell growth. The definitions of early, intermediate and late passages were described above. Cells were treated with/without GR medium as described above. Membranes were reprobed with anti-GAPDH antibody to ensure for equal loading. Representative photographs are derived from three independent experiments.

As shown in Fig. 3A, we found that *p16* mRNA gradually accumulated under the NG condition, whereas GR decreased *p16* transcription as cellular passages progressed in all of the three cell lines we tested. In NG cells, *p16* mRNA accumulated and reached peak levels at the late passage indicating cellular senescence processes were initiated. In contrast to NG treatment, GR resulted in *p16* expression suppression consistently during all cellular passages. However, glucose-restricted IMR-90 cells, which resulted in the longest lifespan extension (66.7%), did not show a maximal reduction of *p16* expression compared with that in WI-38 and MRC-5 cells indicating that although *p16* may serve a major role in lifespan regulation, other factors could also be involved. To elucidate p16 protein expression in response to NG and GR medium, cellular proteins in WI-38 cells were extracted at every other two week intervals and subjected to western blot assay. As indicated in Fig. 3B, p16 protein gradually accumulated from the early five weeks of proliferation in NG growth medium, whereas the p16 protein signal was only detected at the later 12 weeks of treatment with GR medium when cellular replicative senescence may occur. Consistent with *p16* mRNA expression in Fig. 3A, these results suggest that GR-impaired *p16* accumulation may contribute to cellular lifespan extension.

Since *p16* exerts its cellular growth inhibitory effects primarily by negatively regulating Rb phosphorylation, we also examined the *p16*/Rb signaling pathway response to GR. Our results revealed a gradually increased p16 expression was accompanied by reduced expression of phosphorylated Rb (Ser-795) in NG medium during the cellular passaging (Fig. 3C). However, in GR medium, p16 protein signals were barely detected and expression of phosphorylated Rb was increased accordingly (Fig. 3C). Therefore expression alterations of *p16*/Rb signaling were consistent with the model that they are a consequence of changes of cellular PDs and senescence in GR medium suggesting that GR may contribute to increased cellular lifespan via inhibiting the *p16*/Rb signaling pathway. Collectively, these data suggest that GR or CR delays *p16* accumulation and impairs the subsequent *p16*/Rb signaling, which, in turn, inhibits cellular replicative senescence contributing to cellular lifespan extension in normal human cells.

### Over-expressed *p16* resulted in early cellular senescence in glucose-restricted WI-38 cells

**Figure 4:**
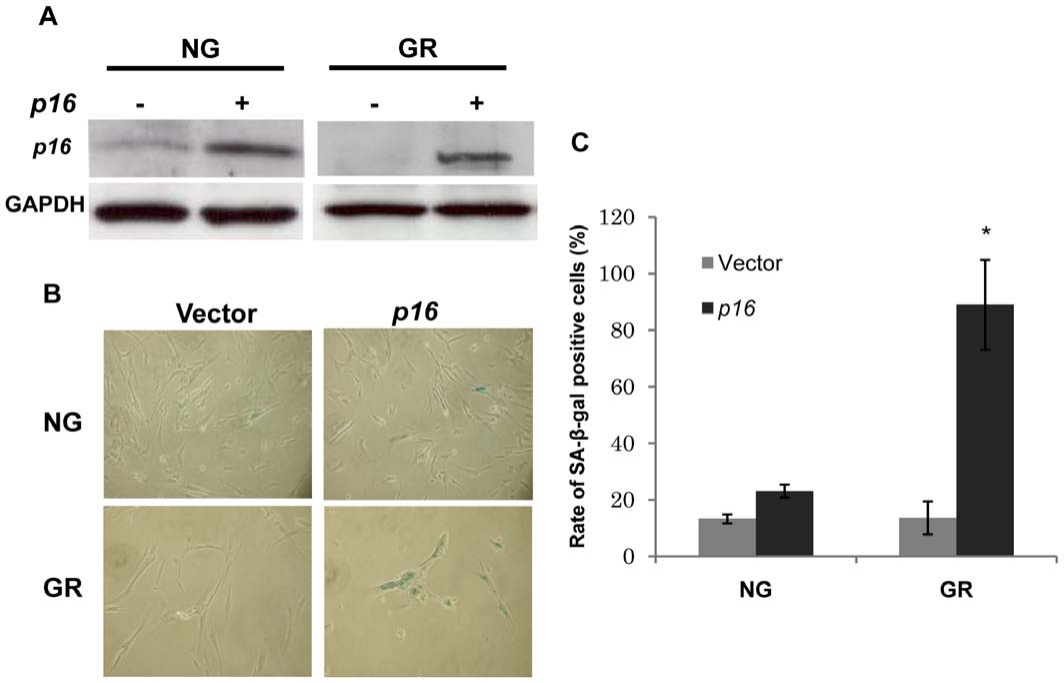
Reversed expression of *p16* accelerated cellular senescence in glucose-restricted WI-38 cells. NG and GR treated WI-38 cells were transiently transfected with empty vector or with cDNA encoding *p16*. A. *p16* expression was verified by western-blot analysis. B. SA-β-Gal assay was used for determining cellular senescence in the post-transfected WI-38 cells. C. The representative graphs showed the rate of SA-β-Gal-positive cells from three independent experiments. Columns, mean; Bars, SD; *, *P*<0.05, significantly different from vehicle control.

To determine the roles of *p16* expression in CR-induced cellular lifespan extension, reversed *p16* expression was obtained by transiently transfecting *p16* into NG or GR-treated WI-38 cells at intermediated passage. p16 protein was detected to verify transfection efficiency and SA-β-activity was performed to investigate cellular senescence as well (Fig. 4A). As shown in Fig. 4B and 4C, *p16* over-expression in GR but not in NG cells caused a significant increase in senescent cells compared with the cells transfected with mock vector in WI-38 cells, which implied that *p16* amount is more critical in senescence regulation under GR rather than NG. These results also suggest that age-impairing properties conferred by *p16* reduction are abrogated when *p16* expression is reversed, which further indicates the importance of *p16* expression in aging regulation during GR.

### GR resulted in chromatin remodeling of the *p16* promoter

Our previous studies have shown different chromatin remodeling patterns of the *p16* gene regulatory region may contribute to different cellular fates in normal and cancer cells in response to GR [15]. We therefore extended our study to investigate the epigenetic mechanisms involving GR-induced dynamic chromatin remodeling in the *p16* promoter in three normal human cell lines. We initiated our experiments to determine chromatin patterns in histone acetylation and methylation due to their important roles in gene regulation. The transcriptional chromatin markers we used included both transcriptionally active (acetyl-H3 and dimethyl-H3K4) and inactive (trimethyl-H3K9) markers.

**Figure 5:**
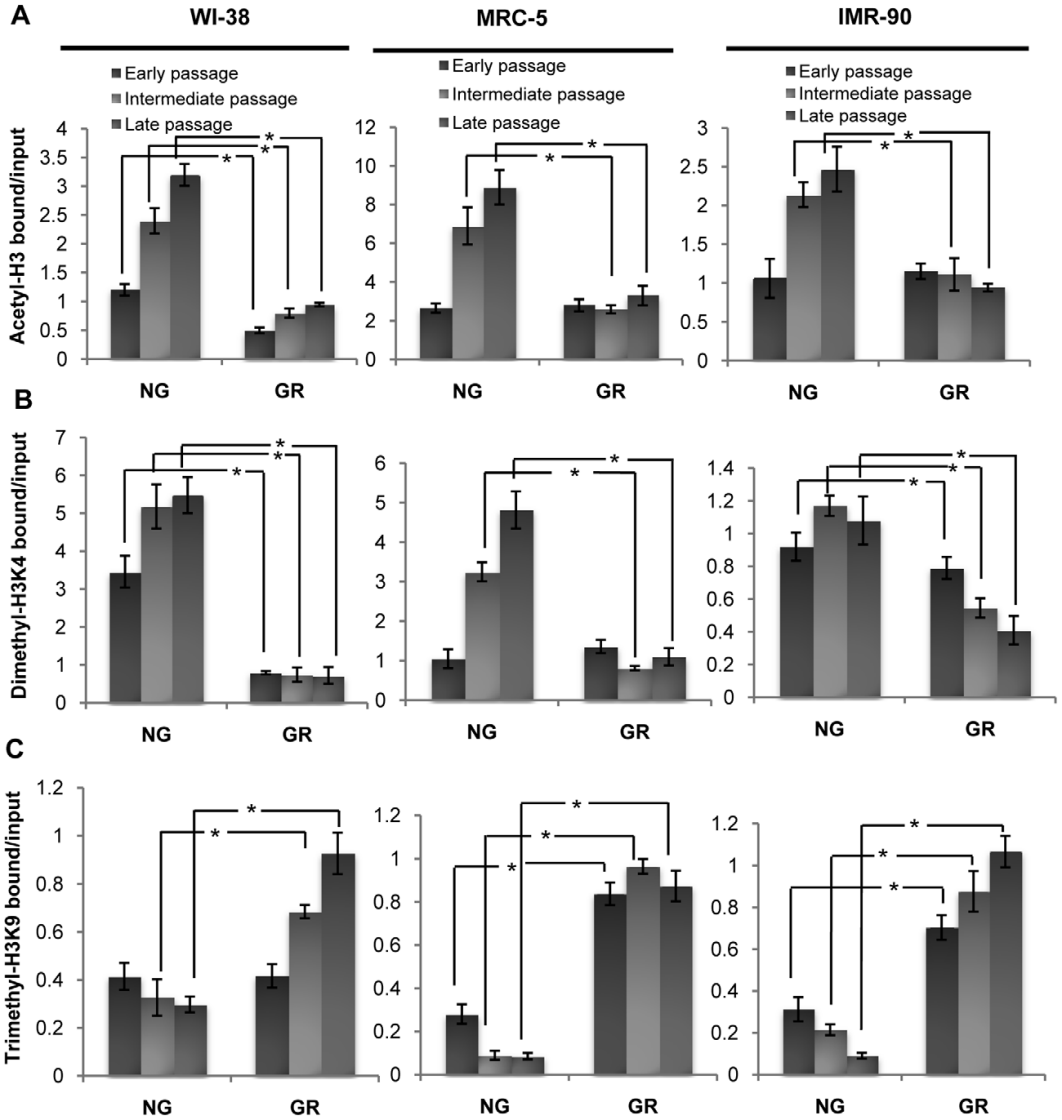
Histone modification changes of the *p16* promoters in response to GR. GR-treated and -untreated human fibroblasts including WI-38 (left), MRC-5 (middle) and IMR-90 (right) were analyzed by ChIP assays. Enrichment changes of three chromatin markers including two activators, acetyl-H3 (A) and dimethyl-H3K4 (B) and one repressor, trimethyl-H3K9 (C), in the *p16* promoter were tested during early, intermediate and late proliferation of cell growth. The relative enrichment of individual chromatin markers was obtained from ChIP PCR and calculated as the ratio between the net intensity of each bound sample divided by the input. Inputs came from the total DNA and served as the same ChIP PCR conditions. Representative photograph from an experiment was repeated three times. Columns, mean; Bars, SD; *, *P*<0.05, significantly different from the corresponding NG control.

We monitored the trend changes of these chromatin markers during cell passage progression to detect the effects of chromatin remodeling on *p16* expression under NG or GR in three tested cell lines. As shown in Fig. 5A (acetyl-H3) and 5B (dimethyl-H3K4), the bindings of these active chromatin markers in the *p16* promoter were significantly decreased by GR compared with NG at most of the cellular passages in all three examined cell lines. By contrast, GR significantly increased the binding of trimethyl-H3K9, the inactive chromatin marker, to the *p16* promoter (Fig. 5C). We also found that GR-induced enrichment changes of these chromatin markers were more significant in the intermediate and late passages than in the early passage suggesting epigenetic mechanisms may involve a metabolic adaptive response via *p16* repression under GR as we discussed previously. It is very feasible that the histone remodeling in the *p16* promoter contributes to *p16* transcription repression by increasing the compaction of histone structures in local DNA replicative loci. More importantly, these chromatin structural changes in the *p16* promoter were consistent with *p16* expression patterns which suggested a strong cause-and-effect relationship between epigenetic mechanisms and *p16* regulation. Taken together, these results suggest that chromatin modification may play a crucial role in CR-induced *in vitro* longevity, at least partly due to epigenetic *p16* repression and that this occurs in all of the cell lines we examined.

### GR-induced SIRT1 expression and enzymatic activity in human fibroblasts

**Figure 6:**
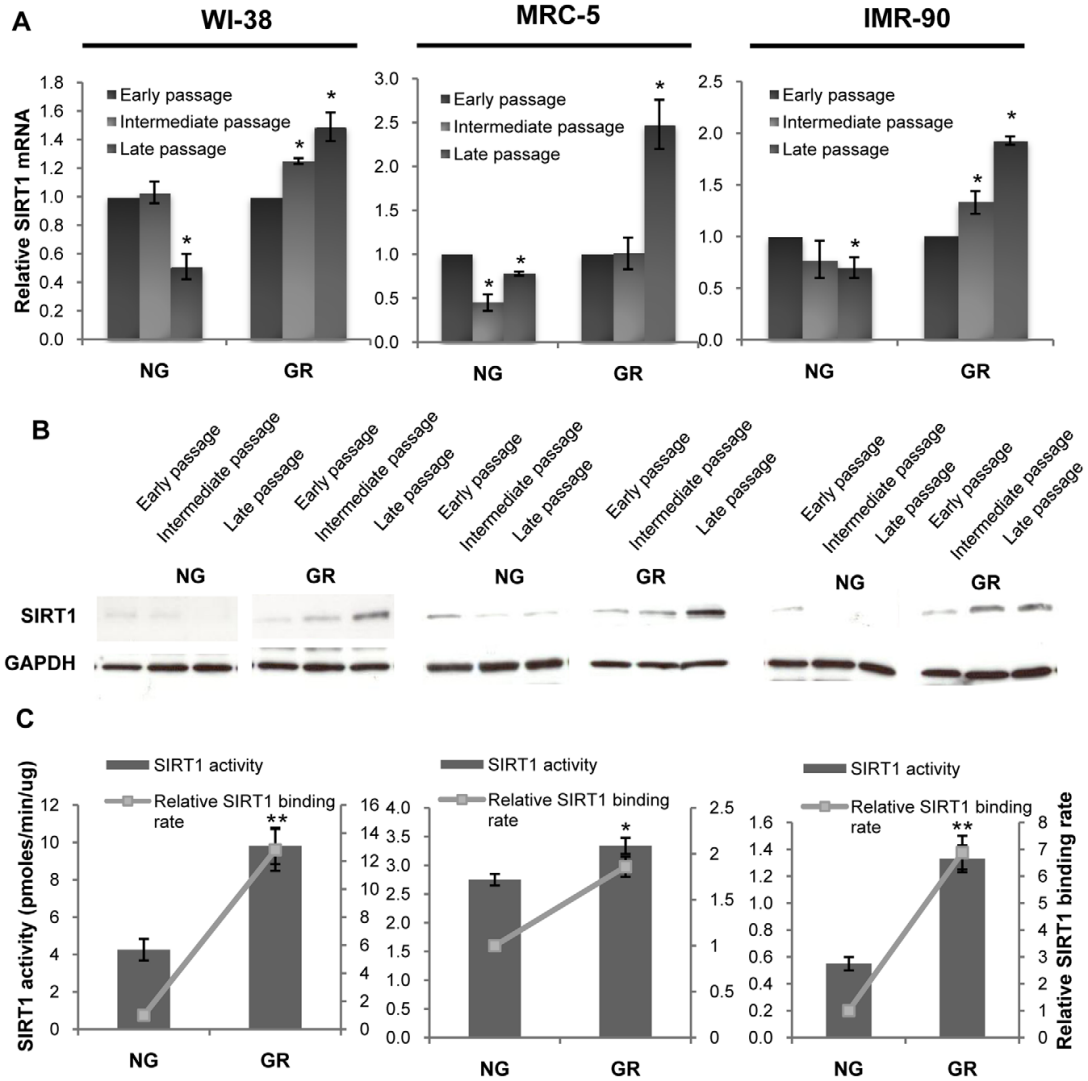
GR-induced activation of SIRT1 expression, HDAC enzymatic activity and binding ability in human fibroblasts. A and B. Expression of SIRT1 in WI-38 (left), MRC-5 (middle) and IMR-90 (right) cells by the treatment of NG and GR growth medium during early, intermediate and late proliferation of cell growth. A, mRNA levels of SIRT1 expression. Representative graphic came from three independent experiments and were normalized to GAPDH and calibrated to SIRT1 mRNA level at PD 0. Columns, mean; Bars, SD; *, *P*<0.05, significantly different from SIRT1 mRNA level at PD 0. B. The protein levels of SIRT1 were determined by western-blot analysis. Representative photographs were obtained from three independent experiments. C. Alterations of SIRT1-HDAC enzymatic activity (columns) and binding ability in the *p16* promoter (lines) in response to GR. Nuclear proteins of human fibroblasts of WI-38 (left), MRC-5 (middle) and IMR-90 (right) cells were extracted at the intermediate progress of cell proliferation. SIRT1 HDAC activity assays were performed according to the manufacturer's protocols. The relative binding ability of SIRT1 to the *p16* promoter was evaluated by ChIP assay and calibrated to NG values. The values of HDAC enzymatic activities and binding enrichments of SIRT1 are the means of three independent experiments. Columns or lines, mean; Bars, SD; *, *P*<0.05, **, *P*<0.01, significantly different from NG control.

Numerous studies have demonstrated the pivotal role of SIRT1 in lifespan prolongation in various species via regulating epigenetic and genetic events. However, although extensive studies have investigated the function of SIRT1 in longevity and metabolic control in animal models, the precise effects of SIRT1 on *in vitro* lifespan extension are not yet known. To determine the roles of SIRT1 in cellular longevity under the circumstance of CR *in vitro*, we analyzed the changes of SIRT1 expression and its histone deacetylase enzymatic activity in human lung fibroblasts in the presence or absence of GR. We discovered that GR significantly increased SIRT1 mRNA (Fig. 6A) and protein levels (Fig. 6B) expression, most notably in late passage of cell growth and that this applied to all of the cell lines we examined. Accordingly, SIRT1 histone deacetylase activities were also found to be elevated significantly and were accompanied by an increased binding of SIRT1 in the *p16* promoter (Fig. 6C). Among three tested fibroblasts, GR-treated IMR-90 cells with the longest lifespan extension have shown a prominent elevated SIRT1 HDAC activity and binding preference in the *p16* promoter indicating that CR-controlled SIRT1 epigenetic regulation may occupy a central position in determination of maximal cellular lifespan. These results strongly suggest that this increased SIRT1 may contribute to cellular lifespan extension and aging impairment, which may regulate age-related genes such as *p16* due to its epigenetic and potentially genetic effects.

### GR resulted in activation of Akt/p70S6K1 signaling

**Figure 7:**
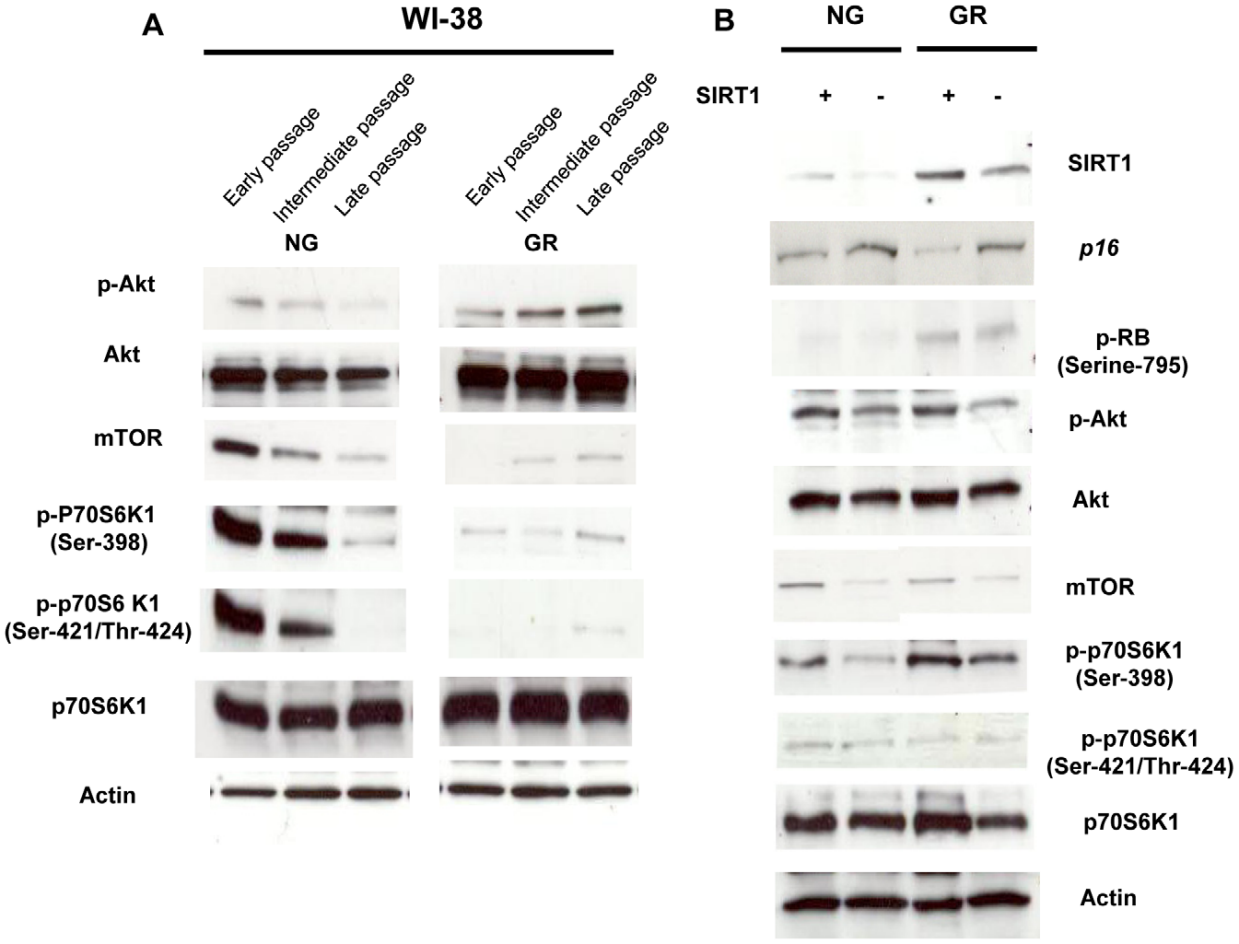
SIRT1 repressed *p16* expression through activation of Akt/p70S6K1. A. Protein expression of Akt/p70S6K1 signaling during early, intermediate and late proliferation of cell growth. Protein from GR-treated or untreated WI-38 cells was transferred to the nitrocellulose membrane and probed with the specific antibodies against Akt, phosphorylated-Akt (Thr-308), mTOR, p70S6K1 and phosphorylated-p70S6K1 (Thr-389, Thr-421/Ser-424). B. Repressed SIRT1 abrogated *p16* inhibition through regulation of Akt/p70S6K1 signaling. GR-treated or untreated WI-38 cells were transfected with SIRT1 siRNA to inhibit SIRT1 expression and extracted protein after three days of transfection. Protein expression alteration of SIRT1, *p16*, p-Rb and Akt/p70S6K1 signaling were detected. Membranes were reprobed with anti-GAPDH antibody to ensure for equal loading. Representative graphic came from three independent experiments.

A large number of publications have reported the importance of Akt signaling on cellular proliferation, metabolism and survival control under nutritional stress [25], [26]. To investigate the precise molecular mechanisms on CR-induced longevity, we examined the Akt kinase pathway and its downstream target, mammalian target of rapamycin (mTOR) in WI-38 cells. As shown in Fig. 7A, phosphorylated Akt and Akt protein kinase showed an increased expression trend during increased PDs in response to GR. Since evidence has shown activation of the Akt pathway induces cell cycle progression due to *p16* repression, our result indicated that GR-induced activated Akt signaling may play an important role in promoting cellular proliferation under the stress of nutrition deficiency via regulation of *p16* expression. The downstream pathway of Akt, mTOR signaling, also plays an important role in longevity in different organisms such as yeast, worms and flies [27]. Mutant mTOR or rapamycin, the inhibitor of mTOR, have been showed to extend the lifespan in yeast and mice [28]. p70S6K1 is a downstream target of Akt and its activation depends on the phosphorylation of several residues of the p70S6K1 protein, such as Ser-389 and Ser-421/Thr-424 [29]. Activation of p70S6K1 is also associated with a decrease in the expression of *p16* [30], [31]. Consistently, our results showed that GR caused a decreased expression of mTOR and its dependent protein kinases, phosphorylated p70S6K1 (Thr-389, Thr-421/Ser-424), whereas it did not affect the expression of p70S6K1 total protein, compared with the protein expression in NG. This suggests that inactivation of mTOR signaling may also contribute to CR-induced longevity *in vitro*. However, a gradient accumulation of mTOR/p70S6K1 protein in GR may result in *p16* silencing due to the enhanced activation of p70S6K1 signaling. In addition, decreased expression patterns in mTOR signaling were found in senescence cells exposed to NG medium indicating the reduction in mTOR/S6K1 signaling may be essential in cellular aging processes (Fig. 7A). Collectively, our results illustrated the important role of Akt/p70S6K1 on cell cycle and senescence regulation and that this effect may be implemented through the inhibition of *p16*, which contributes to lifespan elongation under nutrition stress *in vitro*.

### SIRT1 inhibition abolished Akt/p70S6K1-induced *p16* repression

**Figure 8:**
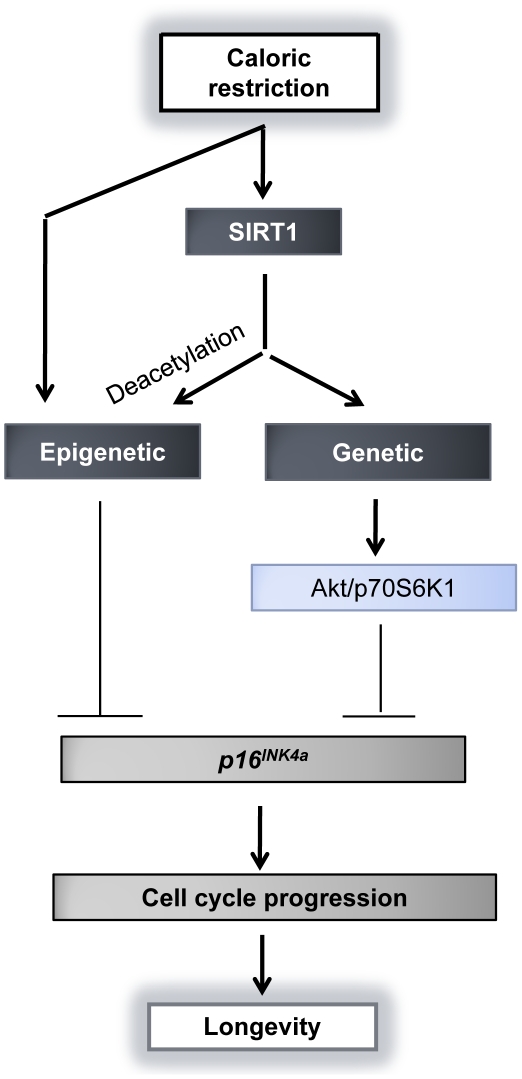
Schematic model for epigenetic and genetic regulation of *p16* repression during CR. CR-induced SIRT1 activation in human lung fibroblasts can suppress the *p16*/Rb pathway through its direct deacetylation effects (epigenetic mechanisms) and indirectly provoking Akt/p70S6K1 signaling (genetic mechanisms). CR can also directly affect epigenetic remodeling in the *p16* promoter which contributes to *p16* repression. As a result, *p16* repression leads to aging delay and cellular lifespan extension.

To further investigate the role of SIRT1 in regulation of *p16* repression, RNAi approaches were performed to repress SIRT1 expression in glucose/glucose restricted-WI-38 cells. As indicated in Fig. 7B, we found that SIRT1 inhibition resulted in reactivation of the *p16*/Rb pathway regardless of nutrition conditions. However, this effect was more efficient and prominent under nutritional stress suggesting that activation of SIRT1 could induce longevity through, at least in part, negative regulation of the *p16*/Rb pathway. We also examined whether SIRT1 suppression can affect the Akt/p70S6K1 signaling pathway, thereby influencing the *p16*/RB pathway. We observed that SIRT1 inhibition abrogated the activation of Akt kinase, mTOR, phosphorylated p70S6K1 (Thr-389, Thr-421/Ser-424) and p70S6K1 (Fig. 7B), suggesting that SIRT1 regulated the *p16*/RB pathway through the Akt/p70S6K1 signaling pathway. In summary, our results revealed a working network involving both epigenetic and genetic control on *p16* regulation under nutrition stress. This model indicated SIRT1 may pose a central role in *p16* repression through direct deacetylation and directly/indirectly modulating Akt/p70S6K1 signaling under the circumstance of nutrition stress (Fig. 8). As a consequence, *p16* repression promotes cellular proliferation and postpones senescence which in turn contributes to lifespan extension and longevity in CR.

## Discussion

Over the past few decades, numerous studies have established that calorie restriction (CR) is the only environmental intervention known to extend maximum lifespan in various species ranging from yeast, worms, flies, mice and even nonhuman primates [1]–[5]. The mechanisms involving the prominent impact of CR on lifespan may be attributed to reduction of the onset of many age-related degenerative diseases, in particular, cancer genesis [6]. At the molecular levels, CR can frequently decrease the expression of age-related genes, thus leading to aging delay and lifespan elongation [9]. Hence CR can extend lifespan mainly through a reduction in the rate of aging [32]. Therefore, studying the alteration and regulation of age-related key genes such as *p16* during CR will not only facilitate exploration of the precise mechanisms involving CR-induced longevity, but also provide inspiration for human aging studies.

In our current studies, we focused on studying the epigenetic and genetic regulation of an important age-related gene, *p16*, in normal human fetal lung fibroblasts during GR. Our results demonstrated that reduced glucose in the culture medium extended cellular lifespan which was accompanied by a reduction in cellular replicative senescence. This increased lifespan was due, at least in part, to *p16* repression by epigenetic chromatin remodeling and activation of SIRT1-mediated epigenetic and genetic regulation. Thus, this study suggests a novel regulation network targeted to epigenetic and genetic control of *p16* expression under the situation of CR and may facilitate an approach to potentially manipulate aging and age-related disease prevention.

Although extensive studies have confirmed that CR can induce longevity in various animal models, evidence is extremely deficient in human studies and *in vitro* studies have not yet been established. We therefore initiated our CR studies in normal human fibroblasts such as WI-38, MRC-5 and IMR-90 cells, by limiting the concentration of their main caloric resource, glucose, in cell culture medium to obtain CR. We found glucose restriction (GR) significantly extended cellular lifespan *in vitro*, which is consistent with previous *in vivo* CR studies [1]–[4]. This extended lifespan correlated with decreased cellular senescence rate suggesting that CR-induced longevity is primarily due to cellular age-delaying effects. Therefore, testing the regulation of important aging control genes during CR may help to understand the mechanisms of CR on aging processes.

Our previous finding showed a differential *p16* expression pattern in normal WI-38 and SV-40 transfected immortalized WI-38 cells may result in different cell fates by which normal cells maintain viability, and the immortalized cells underwent apoptosis in response to glucose deficiency in culture medium [15]. These studies raised the possibility that *p16* regulation is essential in CR-induced nutrient stress adaption and a consequence of longevity. In this study, we therefore examined *p16* expression and found a gradient *p16* accumulation with advancing senescence processes in normal glucose medium, whereas a decreased *p16* expression occurred during CR. Coordinated expression patterns of pRb with *p16* changes further indicated that the effect of CR on aging delay may be due to the suppression of the *p16*/Rb pathway. However, glucose-restricted IMR-90 cells which resulted in the longest lifespan extension (66.7%) did not show a prominent change of *p16* expression among three tested cell lines indicating that *p16* signaling-induced aging postponement may not be the only causative factor although it otherwise correlates with lifespan extension in CR. More importantly, overexpression of *p16* accelerated cellular senescence in GR fibroblasts rather than in NG cells, which further verified the important role of *p16* in CR. It was reported that *p16* acts as a robust biomarker of aging since increased expression of *p16* was observed in almost all the organs in rodents with advancing age [17]. Consistent with those *in vivo* studies, our results suggest that age-associated *p16* accumulation can be attenuated by CR resulting in aging delay and lifespan elongation in cellular aging models.

Although the mechanism connecting this interesting phenomenon is not yet clear, growing evidence showing epigenetic controls on gene regulation and stress modulation led us to speculate that epigenetic mechanisms such as DNA methylation and chromatin modification may play important roles in *p16* regulation in CR [10]–[12]. It has been extensively reported that *p16* expression is frequently regulated by epigenetic events [20], [21]. Our previous studies also indicated that GR-induced DNA hypermethylation of the *p16* promoter impaired E2F-1 binding, thus influencing *p16* expression [15]. Chromatin remodeling including histone acetylation, methylation and phosphorylation, also contributes to *p16* regulation [33]. Here we observed the alteration of chromatin structure was coordinated with *p16* expression patterns in response to GR in all tested cell lines. These results suggest that chromatin modifications influence *p16* expression and also raise a possibility that chromatin-regulation genes such as SIRT1 may also be involved in this process.

SIRT1 belongs to the type III histone deacetylase (HDAC) family and its enzymatic activity is primarily dependent on NAD^+^ (nicotinamide adenine dinucleotide) [34]. SIRT1 is believed to play a crucial role in gene regulation by its epigenetic (chromatin remodeling) and genetic (interacting with a number of transcription factors) effects to obtain stress resistance and longevity under the circumstance of nutrition deficiency [35]–[39]. In this context, SIRT1 is speculated to suppress *p16* expression in a similar manner. This hypothesis was validated by our results showing that GR resulted in a significant increase of SIRT1 expression, its histone deacetylase enzymatic activity and its binding ability to the *p16* promoter as well. In addition, a more significant change in SIRT1 HDAC activity and binding ability to the *p16* promoter was found in GR IMR-90 cells which have the longest lifespan extension suggesting that SIRT1 epigenetic regulation may play a key role in determination of maximal lifespan under CR. This activated SIRT1 may block senescence by direct deacetylation of *p16*, or indirectly impacting certain signal transductions leading to longevity.

We also analyzed the Akt protein kinase signaling pathway not only because the Akt pathway plays major roles in cellular growth, metabolism and aging regulation, but also because Akt activation is associated with *p16* inhibition [25]. In addition, Akt downstream mTOR-p70S6K1 signaling is very important in CR because mTOR acts as a “metabolic checkpoint” in that its activation is tightly dependent on the presence of nutrition [40]. Moreover, activation of the mTOR downstream target, p70S6K1, is reported to be relevant to *p16* repression implying a potential role of p70S6K1 in aging and longevity regulation [31]. Consistent with these reports, our results demonstrated that CR caused activation of Akt/p70S6K1 signaling, indicating these altered signaling pathways conferred a beneficial effect on cell survival and age-delaying through regulation of *p16* expression in response to nutrition deficiency. It was proposed that SIRT1 functions as a nutrient-sensitive growth suppressor gene through regulating multiple signaling pathways including p70S6K1 signaling [14], [31]. Our results confirmed that SIRT1 deficiency recovered the *p16*/Rb pathway accompanied by an attenuation of Akt/p70S6K1 signaling. This suggests that SIRT1 can repress *p16* expression through, at least in part, interacting with Akt/p70S6K1 signaling, leading to longevity in response to CR.

Based on our current study, we propose a potential model where negative regulation of *p16* is mainly mediated by SIRT1 through epigenetic and genetic mechanisms during CR (Fig. 8). SIRT1, acting as a “nutrition sensor”, sensitively responds to environmental nutrition alteration through regulation of various genes that might involve growth control, stress and aging as well. *p16* is known as an important target gene of SIRT1 in a series of physiological and pathological processes including aging and tumorigenesis [16], [17]. According to our theory, CR-induced SIRT1 represses the *p16/*Rb pathway resulting in cell cycle progression, leading to replicative senescence postponement and longevity. *p16* repression was achieved by direct epigenetic silencing though universal chromatin remodeling in the *p16* promoter as well as the increased deacetylation activity of SIRT1, and indirect regulation by interaction with the Akt/p70S6K1 signaling pathway.

Collectively, our findings provide potential mechanisms by which CR leads to aging delay and lifespan extension via regulation of a robust aging biomarker, *p16*, in normal human fibroblasts. More importantly, our results demonstrate a crosstalk model involving SIRT1-mediated epigenetic and genetic regulation on *p16* repression in CR. The important role of CR on aging and age-related diseases such as cancer, has opened an attractive approach to potentially manipulate aging to achieve the ultimate goal of impacting longevity in humans. Our study helps to elucidate the basic mechanisms underlying key gene regulation during nutrition deficiency, which will benefit development of effective therapeutic strategies for aging and age-associated diseases.

## Materials and Methods

### Cell culture and growth kinetics assessment

Normal diploid WI-38, MRC-5 and IMR-90 human fetal lung fibroblasts were obtained from American Type Culture Collection (Manassas, VA, USA). The cells were within the range of 15–20 population doublings (PDs) upon initiation of the experiment. Cells were maintained in DMEM (Invitrogen, Carlsbad, CA, USA) medium supplemented with 4.5 g/L glucose. To restrain glucose, cells were cultured in glucose- and pyruvate-free DMEM medium (Invitrogen). All culture media were supplemented with 10% fetal bovine serum (Atlanta Biologicals, Lawrenceville, GA, USA) and 1% penicillin/streptomycin (Mediatech, Herndon, VA, USA) in the presence of 5% CO_2_ at 37°C. The actual glucose concentration in the glucose restriction medium was 15 mg/L, which was assessed by the Glucose Assay kit (Biovision, Mountain view, CA, USA) following the manufacturer's protocols.

To quantify replication, cells were passaged weekly at a seeding density of 10^5^ cells per plate and counted using a Neubauer haemocytometer until cells reached 90–95% confluence. Cellular PDs were calculated based on the following formula: log [(number of cells harvested)/(number of cells seeded)]/log2 [41]. The first confluent cells while the experiments initiated were designated as PD 0. The duration of cellular lifespan was equally divided in three stages including early, intermediate and late passages. Cellular replicative senescence at the end of late passage in cellular lifespan was determined by morphological changes and senescence-associated β-Galactosidase activity assay as described below. The PD ranges of early, intermediate and late passages in WI-38, MRC-5 and IMR-90 cells with either NG or GR treatment are shown in Table 2.

**Table 2:** Representation of population doublings in early, intermediate and late passages of cell growth in either NR or GR medium.

Cell lines	WI-38			MRC-5			IMR-90		
	Population doublings @			Population doublings @			Population doublings @		
Glucose status	E#	I*	L&	E#	I*	L&	E#	I*	L&
**NG**	0–3	4–6	7–10	0–13	14–27	28–40	0–5	6–11	12–16
**GR**	0–4	5–10	11–15	0–15	16–30	31–45	0–9	10–19	20–27

@ The population doublings were calculated after the first confluent cells designed as PD 0;

# early passage;

* intermediated passage;

& late passage.

### Senescence-associated β-Galactosidase activity assay

The normal human fetal lung fibroblasts (WI-38, MRC-5 and IMR-90) were treated with normal glucose and glucose restriction medium as described preciously [15]. Cellular senescence was assessed by using the Senescence β-Galactosidase staining kit (Cell Signaling, Beverly, MA, USA). In particular, cells were washed twice in phosphate-buffered saline (PBS) and then fixed in 0.5% glutaraldehyde for 10 min at room temperature. After a second wash with PBS, the cells were incubated at 37°C with fresh senescence associated β-Gal (SA-β-Gal) stain solution containing 1 mg/ml of 5-bromo-4-chloro-3-indolyl-beta-D-galactoside (X-Gal) overnight according to the manufacturer's protocols. The senescence-associated β-galactosidase (SA-β-Gal)-positive cells were counted by using a Nikon Coolpix990 digital camera (Nikon, Tokyo, Japan) at 40-fold magnification. Counts were performed at five random locations in the same well and every field was counted three times for determination of the mean percentage of positively stained cells.

### Quantitative real-time PCR

All glucose restriction treated- and untreated-human lung fibroblasts were harvested and total RNA was extracted using the RNeasy kit (Qiagen, Valencia, CA, USA) once a week during the cellular lifespan. Total RNA (5 µg) was reverse transcribed to cDNA using the Superscript II kit (Invitrogen, Carlsbad, CA, USA) with oligo-dT primer. In the real-time PCR step, PCR reactions were performed in triplicate with 1 µg cDNA per reaction and primers specific for *p16* (Hs00923894_ml), SIRT1 (Hs01009006_ml) and glyceraldehyde-3-phosphate dehydrogenase (GAPDH) (Hs99999905_ml) provided by Inventoried Gene Assay Products (Applied Biosystems, Foster City, CA, USA) using the Platinum Quantitative PCR Supermix-UDG (Invitrogen) in a Roche LC480 thermocycler. Thermal cycling was initiated at 94°C for 4 min followed by 35 cycles of PCR (94°C, 15 s; 60°C, 30 s). GAPDH was used as an endogenous control, and normal glucose control was used as a calibrator. The relative changes in gene expression were calculated using the following formula: fold change in gene expression, 2^-ΔΔCt^ = 2^-{ΔCt (Glucose restriction) - ΔCt (Normal glucose control)}^, where ΔCt = Ct (*p16* or SIRT1) - Ct (GAPDH) and Ct represents threshold cycle number.

### Antibodies and immunoblotting

Antibodies against *p16^INK4a^* (Santa Cruz Biotechnology, Santa Cruz, CA), phosphorylated-Rb (Ser-795) (Cell Signaling, Danvers, MA), SIRT1 (Abcam, Cambridge, MA), Akt123 (Cell Signaling), p-Akt123 (Thr-308) (Cell Signaling), mTOR (Cell Signaling), phosphorylated-p70S6K1 (Thr-389, Thr-421/Ser-424) (Cell Signaling) and GAPDH (Santa Cruz Biotechnology) were used in this study.

For western blot analysis, protein extracts were prepared by RIPA lysis buffer (Upstate Biotechnology, Temecula, CA) from normal fetal lung fibroblasts in normal or glucose restricted medium according to the manufacturer's protocol. Proteins (50 µg) were electrophoresed on a 10% SDS-polyacrylamide gel and transferred to nitrocellulose membranes. Membranes were probed with the corresponding antibody, then the membrane was stripped and reprobed with GAPDH as loading control. Molecular weight markers were run on each gel to confirm the molecular size of the immunoreactive proteins. Immunoreactive bands were visualized using the enhanced chemiluminescence detection system (Santa Cruz Biotechnology) following the protocol of the manufacturer.

### Transient transfection of human fetal lung fibroblast WI-38 cells

pcDNA *p16* (expression plasmid) and pcDNA 3.1 (empty vector) plasmids were transiently transfected into NG or GR WI-38 cells at intermediate passage using Lipofectamine 2000 (Invitrogen) according to the manufacturer's protocol. After 72 h post-transfection, cellular protein was extracted and western-blot assays were performed to verify the expression of exogenous *p16* while empty vector transfection served as a negative control. The same batch of cells was subjected to SA-β-Gal staining as described above.

### Chromatin immunoprecipitation (ChIP) assays

Human fetal lung fibroblast cells were cultured in 100 mm tissue culture dishes and harvested at the indicated time points. ChIP assays were performed with the EZ Chromatin Immunoprecipitation (EZ ChIP^TM^) assay kit according to the manufacturer's protocol (Upstate Biotechnology) as reported previously [15]. The antibodies used in the ChIP assays were ChIP-validated acetyl-histone H3 (Upstate Biotechnology), dimethyl-histone H3 (Lys4) (Upstate Biotechnology), trimethyl-histone H3 (Lys9) (Upstate Biotechnology), SIRT1 (Abcam) respectively. In addition, appropriate controls including positive control (RNA polymerase antibody), negative control (mouse IgG) and internal control (input) were established to optimize the ChIP conditions. ChIP-purified DNA was amplified by standard PCR using primers specific for the *p16* promoter. The *p16* ChIP primers were sense, 5′- TAGGAAGGTTGTATCGCGGAGG -3′, and anti-sense 5′- CAAGGAAGGAGGACTGGGCTC -3′ rendering a 172 bp PCR product. PCR amplification was performed using the 2×PCR Master Mix (Promega) and the reaction was initiated at 94°C for 4 min followed by 30 cycles of PCR (94°C, 30 s; 56°C, 30 s; 72°C, 1 min), and extended at 72°C for 5 min. After amplification, PCR products were separated on 1.5% agarose gels and visualized by ethidium bromide fluorescence using Kodak 1D 3.6.1 image software (Eastman Kodak Company, Rochester, NY, USA). Quantitative data were analyzed using the Sequence Detection System software version 2.1 (PE Applied Biosystems, Foster City, CA, USA).

### Histone deacetylase activities of SIRT1

Cultured cells were harvested at the indicated period of treatment, and nuclear extracts were prepared with the nuclear extraction reagent (Pierce, Rockford, IL). The HDAC activity assays (Active Motif, Carlsbad, CA, USA) were performed according to the manufacturer's protocols. NAD (Sigma, St. Louis, MO, USA) was added into the assay buffer to activate SIRT1 activity and nicotinamide served as an inhibitor. The enzymatic activities of HDAC were detected by a microplate reader at 450 nm.

### RNA interference

Validated siRNA for SIRT1 (Santa Cruz Biotechnology) and the appropriate control RNAi (Applied Biosystems) were transfected into WI-38 cells using the Silencer siRNA Transfection II Kit (Applied Biosystems) according to the protocols provided by the manufacturer. To verify the result of SIRT1 gene knockout, western-blot assay was performed using the antibody against SIRT1.

### Statistical analyses

Results from real-time PCR assays were derived from at least three independent experiments. Kodak 1D 3.6.1 image software was used for quantification of ChIP products. Statistical significance between treatment and control groups was evaluated with one-way ANOVA followed by the Dunnett post-test using the Prism3 GraphPad software. Significance level was calculated using an assigned confidence interval of 95%. *P*<0.05 was considered statistically significant.
